# A Miniature System for Separating Aerosol Particles and Measuring Mass Concentrations

**DOI:** 10.3390/s100403641

**Published:** 2010-04-12

**Authors:** Dao Liang, Wen-Pin Shih, Chuin-Shan Chen, Chi-An Dai

**Affiliations:** 1 Department of Mechanical Engineering, National Taiwan University, Taipei 106, Taiwan; E-Mail: daniel@caece.net; 2 Department of Civil Engineering, National Taiwan University, Taipei 106, Taiwan; E-Mail: dchen@ntu.edu.tw; 3 Department of Chemical Engineering, National Taiwan University, Taipei 106, Taiwan; E-Mail: polymer@ntu.edu.tw

**Keywords:** microbalance sensor, virtual impactor, aerosol particle

## Abstract

We designed and fabricated a new sensing system which consists of two virtual impactors and two quartz-crystal microbalance (QCM) sensors for measuring particle mass concentration and size distribution. The virtual impactors utilized different inertial forces of particles in air flow to classify different particle sizes. They were designed to classify particle diameter, *d*, into three different ranges: *d* < 2.28 μm, 2.28 μm ≤ *d* ≤ 3.20 μm, *d* > 3.20 μm. The QCM sensors were coated with a hydrogel, which was found to be a reliable adhesive for capturing aerosol particles. The QCM sensor coated with hydrogel was used to measure the mass loading of particles by utilizing its characteristic of resonant frequency shift. An integrated system has been demonstrated.

## 1. Introduction

The techniques to determine the size distribution and concentration of aerosol particles are of high interest in the field of air quality control [1]. Optical detection is the most common method used to measure particle concentration. A bio-aerosol fluorescence sensor has been developed and was field tested by Reyes *et al.*, and the sensor performed very well in field trials [2]. Davitt *et al.* demonstrated a compact system which incorporates a 32-element linear array of ultraviolet light-emitting diodes for the in-flight fluorescence detection of aerosolized particles. This ultraviolet diode array detected the emission from nicotinamide adenine dinucleotide and tryptophan in aerosol samples with high performance [3]. Ouazzani and Bentama used a photo-interrupt sensor to measure the cake thickness of biological particles [4]. This technique can be applied to the filtration process of a yeast (*Saccharomyces cerevisiae*) suspension. Zemlyanov and Geints reported a method using femtosecond laser pulses to illuminate water droplets, and the scattering effects were then evaluated for calculating the aerosol concentration [5].

Although there have existed numerous promising techniques for measuring aerosol concentration, these developed instruments are often too complicated and bulky for *in-situ* applications. Recently, the miniaturization of the instruments for air quality inspection has become an emerging field. It might provide more functionalities of the system and often decreases costs. In addition to optics, there remain some other methods for detecting particles that can be used in a miniature system. For example, the variation of electric signals can be a good index for counting biological cells, although such a system is always used in fluids [6,7]. In aerosol technology, the so-called virtual impactor which could separate different kinds of particles by their inertial forces is widely used for concentrating aerosols. Haglund and McFarland have developed a circumferential slot virtual impactor to be an aerosol concentrator [8]. The collection efficiency was greater than 72% for all particle sizes larger than three times the cut-off point up to the largest particle size tested, and the peak collection efficiency for the device was greater than 95% [8]. However, their device is still bulky and cannot be integrated into a miniature system. By utilizing micromachining techniques, Lim *et al.* and Maeng *et al.* have successfully miniaturized a cascade virtual impactor [9,10]. Nevertheless, an associated miniature system for directly measuring the mass concentration and size distribution of aerosols is still lacking.

**Figure 1.**
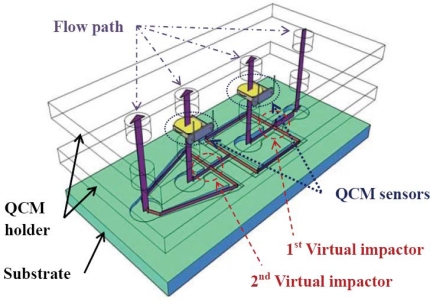
Schematic of virtual impactors integrated with QCM sensors for detecting aerosol particles. The QCM sensors are fixed by the QCM holders. The virtual impactors with channel substructure will separate particles into three different groups of different sizes.

In this paper we propose a miniature system which possesses the functions of separating and concentrating aerosol particles and measuring the mass concentration. Specifically, two virtual impactors are used to separate and concentrate aerosol particles, and two quartz-crystal microbalance (QCM) sensors are used for measuring the mass concentration. The QCM sensor has the advantage of the high sensitivity of its resonance frequency shift in response to mass loading. It also has very short response time and hence is suitable for real-time sensing applications [11,12]. A typical QCM sensor is made of a quartz-crystal disk sandwiched by gold electrodes. If aerosol particles precipitate onto the electrodes, the total mass of the sensing system will increase, causing the resonance frequency drop of the QCM sensor. The frequency shift is more prominent if there are more aerosol particles on the electrodes. Because the aerosol particles usually have a large variety of material compositions, it is difficult to capture aerosol particles properly on the metal electrodes by using specific binding mechanisms. In this paper, physical adhesion is employed to capture aerosol particles on the metal electrodes. To enhance the adhesive force on the metal electrodes, special coating on the surface of the QCM sensor is developed. The miniature system proposed in this paper is shown in Figure 1. It consists of two virtual impactors and two QCM sensors. The system is integrated by embedding the QCM sensors into three-dimensional microchannels which serve as virtual impactors. The virtual impactors separate the particles into three groups of different sizes, and the mass concentrations of two of the groups are measured by the QCM sensors. In this paper the design principles of the virtual impactor will be given first. Then the QCM sensor with the associated coating and characterization method will be introduced. The fabrication of the devices will be given. Then the characterization results of the virtual impactor and the QCM sensor will be shown, respectively. Finally, the integration, testing and verification of the miniature system for separating aerosol particles and measuring the mass concentration will be given.

## 2. Design Principle

### 2.1. Virtual Impactor

The virtual impactor utilizes different inertial forces of particles, which are carried by an air flow, to distribute the particles into two different channels. Hence the flow rate is an important parameter for designing the virtual impactor. The particle cut-off diameter defines the threshold diameter at which the particles are separated into two groups through a virtual impactor. Due to the fact that particles existing in nature are irregular, aerodynamic equivalent diameter is measured by the miniature system in this work [13]. The dimensions of the virtual impactor are dependent on the particle cut-off diameter in accordance to Stoke’s law [14,15]:

(1) $$\mathrm{Stk}=\frac{\tau U}{W/2}=\frac{\rho_{P}d_{P}^{2}{UC}_{C}}{9\mu W}=\frac{\rho_{P}d_{P}^{2}{QC}_{C}}{9\mu hW^{2}}$$

where *Stk* is the Stoke’s number, defined as the ratio of particle stopping distance at the average air velocity (*U*) to half of the virtual impactor width (*W/2*). The symbols *τ*, *ρ_P_*, *d_P_*, *Q*, *μ*, *h*, and *C_C_* denote the relaxation time, the particle density, the particle diameter, the flow rate, the dynamic viscosity of the fluid, the channel height, and the slip correction factor, respectively. The slip correction factor (*C_C_*) is considered only when particle diameter is smaller than 1 μm. *Cc* is one when it is not considered.

**Figure 2.**
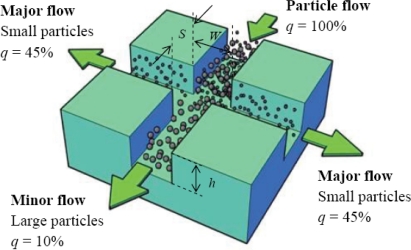
Schematic of the virtual impactor. The large particles follow the minor flow due to larger inertial force. The small particles follow the major flow to the other channels. *q* denotes the percentage of the total flow.

The cross-shaped virtual impactor depicted in Figure 2 is used in this paper. Figure 2 shows that large particles will follow the minor flow due to their larger inertial force. Meanwhile, small particles will follow the major flow to the other two channels because they are not easily affected by their inertial force. Therefore, the particles can be separated into two groups by this virtual impactor. In this work, it is designed that 90% of the particle flow will follow the major flow. The design of the virtual impactor is also related to the Reynolds number, which is given by:

(2) $$\text{Re}=\frac{\rho WU}{\mu }.$$

The Reynolds number is defined as the ratio of the inertial force to the frictional force. For calculating the Reynolds number in the major flow, *W* in Equation 2 should be replaced by *S*. In this work the ratio of the Reynolds number of the major flow to the minor flow is set to be 9.0 so that apparent particle separation effects can be obtained.

Many important parameters should be considered for designing a virtual impactor. Based on the early studies [7–9], the cut-point Stokes number (*Stk*_50_) is set to be 0.23 for designing the rectangular channels in our work. At this designated Stokes number, 50% of the particles with the cut-off diameter will be collected by the virtual impactor. The collection efficiency of the particles with diameters greater than the cut-off diameter will be higher than 50%. After the Stokes number is designated, the flow rate (*Q*), the particle cut-off diameter (*d*_50_), and the channel height (*t*) are decided accordingly. Based on these parameters, the width of the virtual impactor (*W*) can be calculated using Equation 1. Then the Reynolds number (*Re*) is calculated using Equation 2. To obtain reasonable particle collection efficiency, the Reynolds number must be between 500 and 3,000, otherwise, the design parameters of the virtual impactor must be modified. The channel width for the major flow (*S*) is determined based on the injection nozzle width (*W*). It should be noted that the width of the virtual impactor is identical to the injection nozzle width so that the channel interconnection can be simplified in the experiment. For a virtual impactor, the minimum ratio of *S/W* should be larger than 1.5 [7–9]. Through these design procedures, the virtual impactors are designed to provide two cut-off particle diameters to separate particles into three kinds of particle sizes at the total flow rate of 1.0 LPM (liter per min) for potassium-sodium-tartrate (PST) powder. PST powder is used because it is cheap and can be easily produced for the experiment. Under these design conditions, the injection nozzle width (*W*) and the major flow channel width (*S*) are set to be 5,200 μm and 7,800 μm, respectively, in the first virtual impactor. The injection nozzle width (*W*) and the major flow channel width (*S*) are set to be 1,800 μm and 2,700 μm, respectively, in the second virtual impactor. The expected cut-off diameter is 3.15 μm and 2.5 μm, respectively for the first and the second virtual impactor.

### 2.2. QCM Sensor

Quartz crystal is a highly precise and stable resonator and serves as the major component of the QCM sensor. Its resonant frequency changes if any mass is deposited on its electrodes. The relation between the resonant frequency and mass loading can be calculated approximately by:

(3) $$f_{0}+\Delta f=\sqrt{\frac{k}{m_{0}+\Delta m}},$$

where the symbols *f_0_*, *Δf*, *m*_0_, *Δm* indicate the original resonant frequency of the QCM sensor, the frequency shift, the original mass of the QCM sensor, and mass loading on the QCM surface, respectively. Constant stiffness, *k*, of the QCM is assumed.

**Figure 3.**
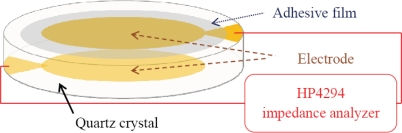
Schematic of a QCM sensor coated with hygrogel adhesive film and the frequency measurement with an HP4294 impedance analyzer. The HP4294 impedance analyzer will send a stable voltage under different frequencies to make the quartz crystal oscillate. The impedance at different frequencies is then measured.

**Figure 4.**
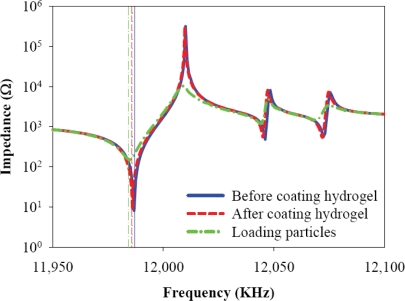
A QCM frequency-impedance test with an HP4294 at three stages: before coating hydro-gel, after coating hydro-gel, and after loading particles. The sharp nodes in the impedance curves represent the resonant frequency points of the QCM sensor. The shift of the first resonant frequency is measured when particles are applied on the surface of the QCM sensor surface.

The QCM sensor has two electrodes. One of the electrodes is on the top surface, and the other is on the bottom surface of the quartz crystal. The electrodes of the QCM sensor are typically made of gold and hence have very poor adhesion to particles. Therefore, the surface of the electrode is modified for improving its particle-capturing capability. Unlike biosensors on which specific binding can be applied, capturing aerosol particles of diverse compositions relies only on physical adhesion. In this work it is found that a hydrogel is a suitable coating layer on the QCM surface to provide sufficient adhesion force for capturing aerosol particles. The preparation of the hydrogel has been detailed by Dong *et al.* [16]. The prepared hydrogel is spun on the electrode with the thickness of ∼400 nm. The hydrogel does not decrease the quality factor (Q-factor) of the quartz resonator, as will be shown in the experiment. A large quality factor would be advantageous for determining the peak response of the resonance signal when the frequency shift of the QCM sensor due to mass loading is measured. Figure 3 shows the measurement setup of the QCM sensor with the adhesive film. An Agilent HP4294 impedance analyzer is used to send excitation voltage under different frequencies to drive the quartz resonator. Meanwhile, the impedance of the QCM sensor at different excitation frequencies is measured. Figure 4 shows one example of the measured impedance of the QCM sensor. The measured first resonant frequency is 11.9867 MHz before the hydrogel is applied on the QCM sensor. After the hydrogel is applied, the first resonant frequency drops slightly to 11.9860 MHz. It should be noted that the resonant amplitude does not have notable change before and after the hydrogel is applied. Hence the hydrogel coating does not degrade the quality factor of the QCM sensor. The high quality factor of the QCM sensor can effectively suppress the signal noise of the measurement [17]. Indeed, the measured impedance in Figure 4 indicates that the signal noise is ignorable. When aerosol particles are applied onto the QCM sensor which has been coated with, hydrogel layer, the resonant frequency drops to 11.9847 MHz, and the resonant impedance increases from 10 Ω to 105 Ω, as shown in Figure 4.

## 3. Fabrication

**Figure 5.**
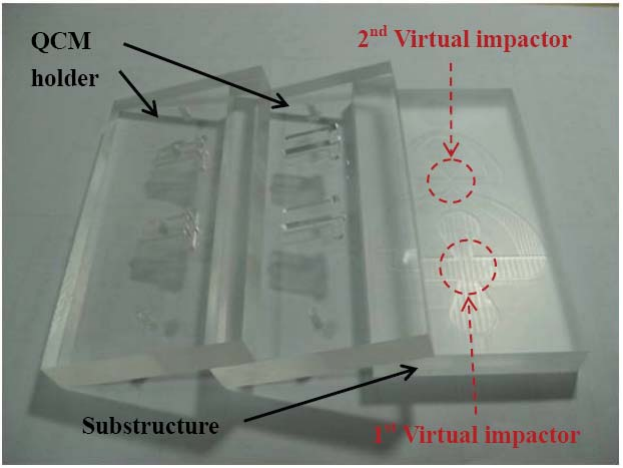
Photograph of the fabricated components.

The virtual impactors are made of bulk polymethylmethacrylate (PMMA). The PMMA material is easy to machine and low-cost. The microchannels and the QCM holders in the virtual impactors are fabricated by using a computer numerical control milling machine. Because the minimum feature size of the microchannels is 1,800 μm, the size of the mill must be smaller than 1,800 μm. A micro grain carbide end mill (tungsten carbide: colt = 9:1 in weight) is used to machine the PMMA material because it is strong enough to sustain long-time machining process without losing precision. In the machining process, all the corners in the virtual impactor are rounded so that the microchannels will not be clogged by aerosol particles. The fabricated components, including the QCM holder and the substructure with two virtual impactors, are shown in Figure 5.

## 4. Experiment

### 4.1. Virtual Impactor

**Figure 6.**
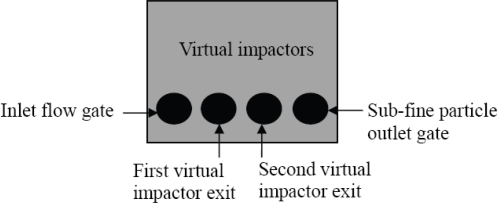
Four gates of the combination of two virtual impactors.

The particle collection efficiency of the fabricated virtual impactors was tested first, before the QCM sensors were implemented. The virtual impactors have four air gates. These four gates, shown in Figure 6, are, from left-to-right, the inlet flow gate, the first virtual impactor exit, the second virtual impactor exit and the sub-fine particle outlet gate, respectively.

**Figure 7.**
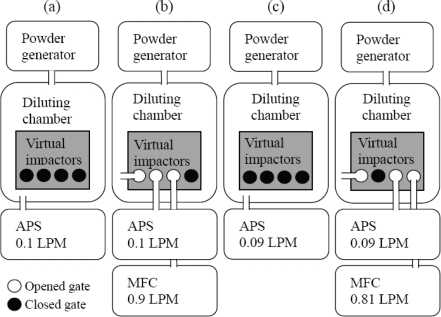
Schematics of the particle collection efficiency experiment of the two virtual impactors. The particle size distribution at each step is measured with an aerodynamic particle sizer.

The test is conducted with a powder generating system. Specifically, the fabricated virtual impactors are placed inside a diluting chamber, in which the particles injected by the powder generator are diluted. The diluting chamber is a rectangular box with 0.6 × 0.6 × 1 m^3^. An aerodynamic particle sizer (APS, TSI, USA). is employed to determine particle distributions. For determining the particle distribution generated by the powder generator, we firstly use the APS with the flow rate of 0.1 LPM to measure the particle distribution of the environment in the diluting chamber, as shown in Figure 7(a). Then we connect the inlet flow gate to the diluting chamber, the first virtual impactor exit to the APS with the flow rate of 0.1 LPM, and the second virtual impactor exit to a mass flow control (MFC) with the flow rate of 0.9 LPM, as shown in Figure 7(b). The sub-fine particle outlet gate is closed at this step. The particle distributions obtained with the APS in Figure 7(a,b), respectively, are then compared to evaluate the particle collection efficiency in the first virtual impactor. For a single particle diameter, the collection efficiency is defined the number ratio of the particles retained inside the virtual impactor to those injected by the powder generator.

**Figure 8.**
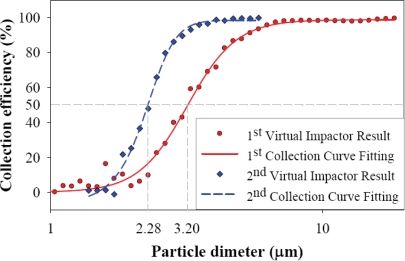
Results of the virtual impactor experiment. The 50% collection efficiency indicates the cut-off particle diameter of the fabricated virtual impactors.

To evaluate the particle collection efficiency in the second virtual impactor, we firstly measure the particle distribution in the diluting chamber with the APS with the flow rate of 0.09 LPM, as shown in Figure 7(c). We then connect the inlet flow gate to the diluting chamber, the second virtual impactor exit to the APS with the flow rate of 0.9 LPM, and the sub-fine particle outlet gate to the MFC with the flow rate of 0.81 LPM, as shown in Figure 7(d). It should be noted that the sub-fine particle outlet connects to the major flow of the second virtual impactor. Hence it is closed for testing the collection efficiency of the first virtual impactor. When the second virtual impactor is being characterized, the flow rate at the sub-fine particle outlet should be controlled. The first virtual impactor exit is closed at this step. Finally, the particle distributions obtained with the APS in Figure 7(c,d), respectively, are compared. The results are shown in Figure 8. The two collection curves are fitted to the raw data obtained with the APS, and the cut-off diameters of the two virtual impactors are 3.20 μm and 2.28 μm, respectively.

### 4.2. QCM Sensor

The characteristic function of the QCM sensor coated with hydro-gel is determined by measuring the resonant frequency shift using an HP4294 impedance analyzer with different particle loadings. The total mass of the loaded acrylic particles on the QCM sensor is estimated by counting the particle numbers from optical images. The characteristic function in Equation (3) can be rewritten in a linear form as:

(4) $$\frac{1}{{\left(f_{0}+\Delta f\right)}^{2}}=\frac{m_{0}}{k}+\frac{\Delta m}{k}=a+b\Delta m$$

The coefficients *a* ≡ *m*_0_ / *k* and *b* ≡ 1 / *k* are independent of the mass loading effects and describe the characteristic function. To determine the coefficients *a* and *b*, we firstly coat the QCM sensor with hydrogel to improve the surface adhesion. Then the resonant frequency of the QCM sensor is measured, followed by applying acrylic particles on the hydrogel surface. After taking optical images of the hydrogel surface, we count the total number of the acrylic particles in these images using an image processing program. Meanwhile, the resonant frequency shift after loading different numbers of acrylic particles is measured.

**Figure 9.**
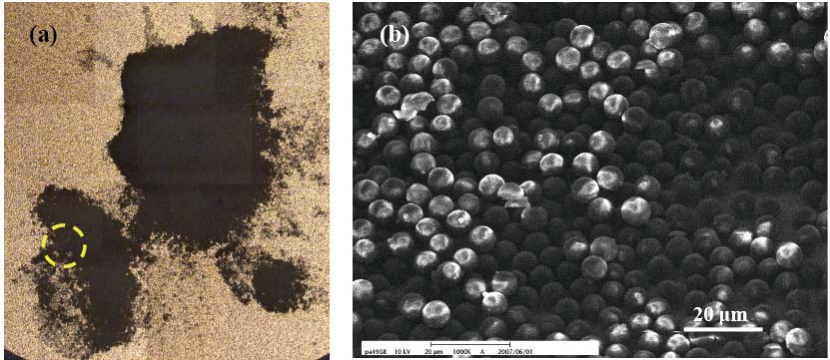
(a) Image of the surface of the QCM sensor taken under an optical microscope. The black regions are particles attached on the hydrogel coating. (b) SEM image of the particles in the circled region in (a). It indicates that the acrylic particles would aggregate with more than one layer on the QCM sensor surface.

**Figure 10.**
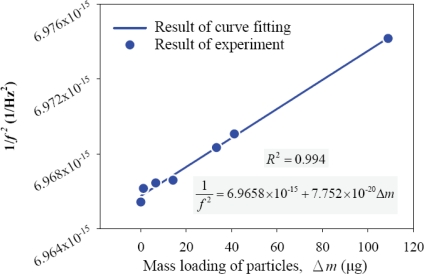
The measured characteristic function of the QCM sensor.

It should be noted that the loading weight on a single QCM sensor is too small to be measured using a microbalance. Therefore, an image processing method is employed. Specifically, every single pixel in the image represents a certain number of particles. An image of the particles on the hydrogel layer taken with an optical microscope is shown in Figure 9(a). In additional to optical images, the particles captured by the hydrogel layer are inspected with a scanning electron microscope (SEM), as shown in Figure 9(b). It is found that the particles on the hydrogel may aggregate into more than one layer. These multi-layers are difficult to be observed under optical microscope. In this work, we identify these multi-layers zones through the SEM inspection and then modify the results of the image processing. Each particle with 10 μm in diameter has the volume of 523.6 μm^3^, and the density of the acrylic particle is 1,190 kg/m^3^. Hence the weight of each acrylic particle is about 0.6 ng. For an area of 1 cm^2^ which is occupied by single-layered particles, the mass loading is approximately 76.4 mg. The dots in Figure 10 show the measured correlation between the resonant frequency of the QCM sensor and the mass loading of the particles. A linear relation between 1/*f*^2^ and Δ*m* is obtained, as predicted by Equation (4). By fitting Equation (4) into the results of experiment, the characteristic function of the QCM sensor with *a* = 6.9658 × 10^−15^ Hz^−2^ and *b* = 7.752 × 10^−20^ Hz^−2^μg^−1^ is obtained. The square of Pearson product-moment correlation coefficient of this fitted curve is R^2^ = 0.994.

### 4.3. Integrated Virtual Impactors and QCM Sensors

**Figure 11.**
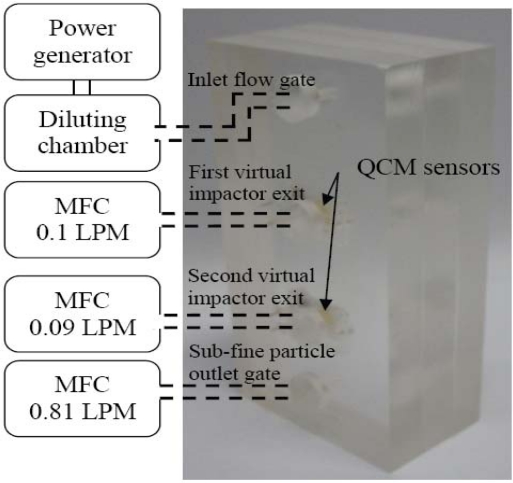
The QCM sensors are put into the virtual impactors for testing the integrated system. The interconnections between the virtual impactors and the flow controllers are illustrated by dashed lines.

**Figure 12.**
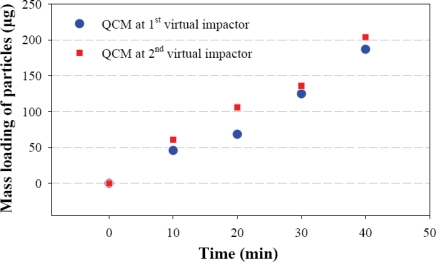
Measured mass loading of the QCM sensors with respect to time.

Two QCM sensors were next integrated into the fabricated virtual impactors for system-level testing. In this test, the inlet flow gate is connected to the diluting chamber. The first virtual impactor exit, the second virtual impactor exit, and the sub-fine particle outlet gate are connected to MFC with flow rate of 0.1 LPM, 0.09 LPM, and 0.81 LPM, respectively as shown in Figure 11. We measure the resonant frequency of the two QCM sensors every ten minutes when particles are injected into the diluting chamber. The characteristic function in Equation (4) with *a* = 6.9658 × 10^−15^ Hz^−2^ and *b* = 7.752 × 10^−20^ Hz^−2^μg^−1^ is then used to calculate the mass loading particles on the QCM sensors. The testing result in Figure 12 indicate the mass loading on both of the QCM sensors increases linearly with time. It implies that the QCM sensors dynamically response to the mass concentration as particles are continuously injected into the diluting chamber. The total volume of the air passing through the virtual impactors can be calculated by multiplying the constant flow rate with the particle injection time. Hence the mass concentration in the air can be obtained by dividing the measured mass loading with the air volume. Since the first and the second virtual impactors have different cut-off diameters of particles, the mass concentrations of different particle sizes are obtained. The mass concentrations measured by the integrated system are given in Table 1. The integrated system can effectively separate the particles into different groups and determine the mass concentrations.

## 5. Discussion and Conclusions

The measured particle collection efficiency indicates that the cut-off particle diameters of the fabricated virtual impactors are 3.2 μm and 2.82 μm, respectively. In this aspect, the fabricated virtual impactors can separate particles into three groups of different sizes. The measured cut-off diameters deviate slightly from the designed values. This discrepancy could be caused by the asymmetric flow in the particle deposition, as implied in Figure 9. The asymmetric flow could be due to the machining precision or the assembly of QCM sensors and microchannels.

To determine the characteristic function of the QCM sensors, we assumed that the inverse square of the resonant frequency is linearly proportional to the mass loading and that the stiffness of the QCM sensor is constant. When the mass loading increases from zero to 100 μg as shown in Figure 10, the inverse square of the resonant frequency varies by only 0.17%, which justified our linear model. In addition, the experiment fits the linear model very well. The maximum mass loading in the system-level test is 200 μg, as shown in Figure 12. Hence the same characteristic function can be applied for determining the mass concentration.

The precision for measuring the mass concentration depends on the minimum detectable mass, flow rate and testing duration. In the calibration in Figure 10, the minimum mass loading we have detected is 0.83 μg. For the 0.1 LPM flow rate and 40 minute testing duration, the measurement precision is 0.208 mg/m^3^. If the testing duration reduces to 1 minute, the measurement precision becomes 8.320 mg/m^3^. Nevertheless, this precision is sufficient to measure the mass concentration in this work.

In summary, we designed, fabricated, and tested a miniature aerosol sensing system. The virtual impactors successfully separated particles into three particle groups of different diameters. And the integrated QCM sensors and the virtual impactors have been demonstrated to be able to determine the mass concentration of different particle groups. The demonstrated system can be potentially applied to air quality monitoring in the future.

## Figures and Tables

**Table 1.** Measured mass concentration of the integrated system.

**Particle size**	**Mass concentration (mg/m^3^)**
>3.20	42.095
3.20∼2.28	52.553

## References

[1] Sun Z., Huang Z., Wang J.S. (2006). Studies on the size distribution, number and mass emission factors of candle particles characterized by modes of burning. J. Aerosol Sci.

[2] Reyes F.L., Jeys T.H., Newbury N.R., Primmerman C.A., Rowe G.S., Sanchez A. (1999). Bio-aerosol fluorescence sensor. Field Ana. Che.Techn.

[3] Davitt K.M., Song Y.K., Patterson W.R., Nurmikko A.V., Pan Y.L., Chang R.K., Gherasimova M., Han J., Cobler P.J., Butler P.D., Palermo V., Gaska R. A compact aerosol sensor and spectroscopic sorting with UV LEDs.

[4] Ouazzani K., Bentama J. (2007). A promising optical technique to measure cake thickness of biological particles during a filtration process. Desalination.

[5] Zemlyanov A.A., Geints Y.E. (2007). Aerosol scattering of supercontinuum radiation formed upon femtosecond laser pulse filamentation in the atmosphere. Opt. Commun.

[6] Satake D., Ebi H., Oku N., Matsuda K., Takao H., Ashiki M., Ishida M. (2002). A sensor for blood cell counter using MEMS technology. Sens. Actuators B.

[7] Tanabe R., Hata S., Shimokohbe A. (2006). MEMS complete blood count sensors designed to reduce noise from electrolysis gas. Microelectron. Eng.

[8] Haglund J.S., McFarland A.R. (2004). A circumferential slot virtual impactor. Aerosol Sci. Techn.

[9] Lim H.H., Park D., Maeng J.Y., Hwang J., Kim Y.J. MEMS based integrated particle detection chip for real time environmental monitoring.

[10] Maeng J.Y., Park D., Kim Y.H., Hwang J., Kim Y.J. Micromachined cascade virtual impactor for aerodynamic size classification of airborne particles.

[11] Tzou T.Z. (1999). Aerodynamic particle size of metered-dose inhalers determined by the quartz crystal microbalance and the Andersen cascade impactor. Int. J. Pharm.

[12] Nakamoto T., Suzuki Y., Moriizumi T. (2002). Study of VHF-band QCM gas sensor. Sen. Actuators B.

[13] Timbrell S.T. (1965). Human exposure to asbestos: dust controls and standards. The inhalation of fibrous dusts. Ann. N. Y. Acad. Sci.

[14] Hounam R.F., Sherwood R.J. (1965). The cascade centripeter: a device for determining the concentration performance. Am. Ind. Hyg. Assoc. J.

[15] Lim K.S, Lee K.W. (2006). Collection efficiency and particle loss of virtual impactors with different methods of increasing pressure drop. J. Aerosol Sci.

[16] Dong L., Agarwal A.K., Beebe D.J., Jiang H.R. (2006). Adaptive liquid microlenses activated by stimuli-responsive hydrogels. Nature.

[17] Rodríguez-Pardo L., Rodríguez J.F., Gabrielli C., Perrot H., Brendel R. (2005). Sensitivity, noise, and resolution in QCM sensors in liquid media. IEEE Sens. J.

